# Mathematical Modelling of Residual-Stress Based Volumetric Growth in Soft Matter

**DOI:** 10.1007/s10659-021-09834-8

**Published:** 2021-05-20

**Authors:** Ruoyu Huang, Raymond W. Ogden, Raimondo Penta

**Affiliations:** 1 Lightweight Manufacturing Centre, University of Strathclyde, Renfrew, PA4 8DJ UK; 2 School of Mathematics and Statistics, University of Glasgow, Glasgow, G12 8QQ UK

**Keywords:** Residual stress, Volumetric growth, Nonlinear elasticity, 74B20, 74L15

## Abstract

Growth in nature is associated with the development of residual stresses and is in general heterogeneous and anisotropic at all scales. Residual stress in an unloaded configuration of a growing material provides direct evidence of the mechanical regulation of heterogeneity and anisotropy of growth. The present study explores a model of stress-mediated growth based on the unloaded configuration that considers either the residual stress or the deformation gradient relative to the unloaded configuration as a growth variable. This makes it possible to analyze stress-mediated growth without the need to invoke the existence of a fictitious stress-free grown configuration. Furthermore, applications based on the proposed theoretical framework relate directly to practical experimental scenarios involving the “opening-angle” in arteries as a measure of residual stress. An initial illustration of the theory is then provided by considering the growth of a spherically symmetric thick-walled shell subjected to the incompressibility constraint.

## Introduction

From the nanoscale to the organ scale mechanical designs play important roles in natural soft materials for maintaining particular strength, rigidity, stability, pattern, shape and functionality [16, 17, 20, 35]. The designs are fulfilled by growth, which is an evolving biological process of gene expression regulated by both chemical and mechanical mechanisms. The growth mechanisms in plants and animals are different. The former exhibits the existence of a threshold of stress, i.e. a yield stress, as the necessary condition for growth [7, 19, 27, 32]. By contrast, in the latter a destiny stress plays a key role, this being referred to as a homeostatic stress [16] (or, variously in the literature, as preferred stress, target stress, optimal stress, or growth-equilibrium stress). The scope of the present study is restricted to the growth of animal soft tissue.

The proposal of Fung [16] that homeostasis is an optimal state is widely accepted and is indicative of the role of mechanics in soft tissue growth. The generation of opening angles from an unloaded homeostatic artery wall highlights the importance of residual stresses. The relationship between homeostasis and residual stresses has been a crucial issue in the biomechanics of soft tissue for the last two decades [5, 22, 42]. It can be studied from different points of view, which may give useful insight into two key aspects of growth, i.e. heterogeneity and anisotropy.

Fung [16] proposed a one-dimensional stress–growth law in the form

1 $$ \dot{M}=C (s-a)^{k_{1}}{(b-s)}^{k_{2}}(s-c)^{k_{3}}, $$

where $\dot{M}$ is the rate of change of the volume of the tissue, $s$ is the stress, $a$, $b$, $c$, with $c< a< b$, are constants having units of stress and corresponding to three homeostatic states in sequence, $C$ is a constant, and $k_{1}$, $k_{2}$, $k_{3}$ are positive dimensionless numbers. According to equation (1), tissue starts to grow when the stress increases from its steady state value $a$, while resorption occurs if stress increases sufficiently beyond the value at the higher homeostatic stress $b>a$. Fung’s equation (1) thus illustrates his two basic ideas: (i) growth can be stress-driven; (ii) growth cannot be unbounded with respect to stress.

The generalization of (1) to three-dimensional models can be classified roughly into two types of models. The first is the class of kinematical growth models that predict how kinematical change can regulate growth to achieve homeostasis. The second is the class of material-regulation growth models that predict how the change of mechanical properties via mass change can also regulate growth for the same purpose. The co-existence of the two different classes of models, which has been noticed by some researchers [36, 48], indicates the complexity of growth since, although a certain homeostasis can be achieved by either class of models, the interpretation, modelling and resulting residual stress can differ significantly.

In the context of continuum mechanics, a kinematic theory of growth was first proposed by Skalak [46] and co-workers [47] and given a general formulation by Rodriguez et al. [44] based on the multiplicative decomposition of the deformation gradient into growth and elastic parts, some details of which will be discussed in Sect. 2.

On one hand, kinematic growth models can be formulated directly based on experimental findings of homeostasis without reference to thermodynamic constraints [15, 43, 50]. On the other hand they can be established based on thermodynamics concepts such as the Eshelby stress without necessarily relating to homeostasis [2, 10, 14]. Variational principles are also useful tools for presenting models of growth consistent with thermodynamics concepts [34, 39].

Based on Fung’s mass-stress relation (1), Humphrey, Rajagopal and co-workers [25, 51] developed a series of growth and remodelling (G&R) models that differ from the kinematic growth models. They argued that, although it had achieved great success, kinematic growth has limitations for the understanding of G&R, whereas their G&R models refer more explicitly to the role of the constituents. The multi-species open system in their G&R model naturally refers to mixture theory, which was also studied by other researchers, such as Garikipati et al. [18], Quiligotti [41] and Preziosi and Vitale [40] in the context of growth. A rigorous thermodynamic analysis of an open system was proposed by Kuhl and Steinmann [30] and is consistent with the adaptive elasticity proposed by Cowin and Hegedus [8]. For reviews from different perspectives we refer to Ambrosi et al. [1] and Menzel and Kuhl [37].

Growth is varied and complex, and there is no universal growth model for all tissues, but this does not mean that it is impossible to assess a model. The mechanisms of growth for specific tissues, once determined, will allow more informed model developments, and enable discrimination between the existing competing classes of models.

Residual stresses provide key quantitative evidence for understanding the mechanical mechanisms of growth in the sense of either opening angles or surface wrinkles, folds and creases due to growth-induced buckling and instability [28, 29, 31]. Therefore, from the modelling point of view, it is of interest to consider residual stress explicitly as a proxy for growth rather than as a consequence of growth.

A key challenge is that the connection between residual stresses and growth is still far from being fully understood. Firstly, how residual stresses are generated may have different interpretations, such as kinematic incompatibility or differences between the natural configurations of the constituents. In this connection we quote from the paper by Humphrey and Rajagopal [26] ‘... the concept of natural configuration should be considered both locally and individually for each constituent...’, which indicates that constituents having the same natural states may exhibit kinematic incompatibility. Secondly, whether or not newly deposited materials are stress free and how they affect the residual stress in tissues are still open questions, which can be crucially relevant for industrial and/or biomimetic applications; see, for example, Zurlo and Truskinovsky [52]. Thirdly, the interplay between residual stress and heterogeneity/anisotropy of growth needs to be further clarified. In fact, in the past few years there have been several innovative attempts dealing with inhomogeneities and conservation laws associated with growth, such as [11, 21]. Fourthly, the extent to which residual stress can regulate growth is not clear. The study of residual stresses may therefore shed new light on the mechanics of growth.

In order to set the scene for a modelling strategy that explicitly incorporates residual stress in the growth law, it is worth comparing the experimental evidence for both metal plasticity and soft tissue growth. Plasticity theory, involving a so-called intermediate configuration, was proposed mainly based on the uniaxial tensile test, which directly provides data for the plastic strain as the internal variable. Moreover, the intermediate configuration is directly connected to metal crystal theory. As shown in crystal plasticity, anisotropic plastic flow can be obtained directly from the micromechanical modelling of crystals, as, e.g., the slip system described by the Schmid tensor [3]. Thus, the intermediate configuration has both a micromechanical explanation and the support of experimental data from a tensile test.

However, at present, in the theory of growth, the (unstressed) intermediate configuration does not explicitly link to any micromechanical information that relates to the *anisotropy of growth*, similar to that of the Schmid tensor in plasticity theory. Instead, the existing models of anisotropic growth were mainly based on macroscopic *anisotropy of material* [33, 36]. These macroscopic anisotropic models cannot always encode anisotropic growth that is postulated to have different types of regulation in different directions [4, 49], although there are some instances where this is examined; see [38] for an example relevant to a specific class of distortions in cylinders where the interplay between inhomogeneities and anisotropy is addressed. One example of the complexity of anisotropy is the helical architecture of fibres where material symmetry alone may not suffice to account for the anisotropy of growth [12, 24]. Therefore, the notion of an intermediate configuration may be more relevant to plasticity than to growth from the point of view of experiment observation.

One of the essential issues for exploiting Fung-type opening angle experiments may be the choice of an experimentally more relevant configuration for establishing a growth model. We emphasize that the grown (intermediate) configuration is stress free and does not represent a realistic geometry as it is a set of imaginary, in general non-accessible, local neighbourhoods. On the other hand, the unloaded configuration contains two key pieces of information about growth, i.e. the geometry and the residual stress.

However, unlike the plastic strain in the unloaded configuration of plasticity, the residual stress or its measurement (via, e.g., the opening angle), has rarely been used explicitly as a variable in a time-dependent growth model. This situation leaves a disconnect between modelling and experimental observation.

The purpose of the present study is to establish a framework for growth modelling based on the experimentally more accessible unloaded configuration. By presenting such a framework, the present study shows that the growth variable can be either the geometry of, or the residual stress in, the unloaded configuration. Thus, a growth model based on the unloaded configuration may provide an appropriate phenomenological model so that the interpretation of the sources of residual stresses becomes unnecessary, which would avoid the danger that a growth model may be established on disputable grounds. Moreover, there is no need to assume a pre-stressed reference configuration (before growth), as is sometimes proposed, for exploiting evidence of growth, for example surface buckling, while explicitly or implicitly retaining the stress-free grown configuration. See, for example, [6, 13] for an approach based on a multiplicative decomposition of the deformation gradient.

The present work is organised as follows. First, in Sect. 2, a decomposition of the deformation gradient is proposed that does not involve a fictitious intermediate configuration but, instead, the unloaded configuration, as distinct from the Rodriguez et al. [44] decomposition. Secondly, free energy functions defined relative to the unloaded configuration are presented that consider either the residual stress or the deformation gradient in the residually-stressed configuration (relative to a fixed initial reference configuration) as a structure tensor/growth variable. The associated constitutive constraints are discussed as an aid to understanding the elastic constitutive laws, growth laws and the associated dissipation.

In Sect. 3, growth of a spherically symmetric shell is studied as a first application of the theory in order to illustrate the modelling relative to the unloaded configuration and the connections with the conventional kinematical growth model.

Finally, some discussion and concluding remarks are provided in Sect. 4.

## Elastic Constitutive Laws

### Kinematics

Suppose that at some initial time an intact tissue is represented as a continuum by some fixed reference configuration, $\mathcal{B}_{0}$, with material points labelled by the position vector $\mathbf{X} \in \mathcal{B}_{0}$. At any subsequent time $t \in \left [ {0, \infty } \right )$ the tissue, after having been subject to growth and the application of mechanical loads, occupies the configuration ℬ, which is related to $\mathcal{B}_{0}$ through the mapping $\boldsymbol{\chi } :\mathcal{B}_{0} \to \mathcal{B}$, both configurations being embedded in the Euclidean space $\mathbb{R}^{3}$. This defines the motion or deformation of the tissue, and the current position of a material point $\mathbf{X}$, denoted $\mathbf{x}$, is written as $\mathbf{x} = \boldsymbol{\chi } (\mathbf{X},t) \in \mathcal{B} \subset \mathbb{R}^{3}$.

The associated deformation gradient is denoted $\mathbf{F}$ and defined by $\mathbf{F}(\mathbf{X},t) = \operatorname {Grad}\boldsymbol{\chi }$, where $\operatorname {Grad}$ is the gradient operator with respect to $\mathcal{B}_{0}$. The Jacobian of the deformation, denoted $J\, (>0)$, is given by $J = \det \mathbf{F}$.

**Fig. 1:**
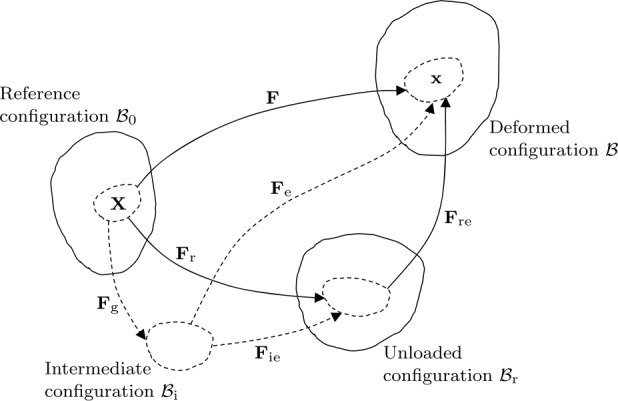
Schematic of the reference, unloaded, deformed and intermediate configurations, $\mathcal{B}_{0}$, $\mathcal{B}_{\mathrm{r}}$, ℬ and $\mathcal{B}_{\mathrm{i}}$, respectively, with their connecting deformation gradients. Note that in general $\mathbf{F}_{\mathrm{e}}$, $\mathbf{F}_{\mathrm{g}}$ and $\mathbf{F}_{\mathrm{ie}}$ (identified by dashed curves) are not gradients of deformations because $\mathcal{B}_{\mathrm{i}}$ is not in general an intact configuration. On the other hand, $\mathbf{F}_{\mathrm{r}}$, $\mathbf{F}$ and $\mathbf{F}_{\mathrm{re}}$ (identified by continuous curves) are gradients of deformations

When the mechanical loads are removed from the tissue it relaxes elastically from ℬ to an unloaded, but in general residually stressed and intact, configuration, which is denoted $\mathcal{B}_{\mathrm{r}}$, as indicated in Fig. 1. The associated deformation gradient that would take $\mathcal{B}_{\mathrm{r}}$ back to ℬ is denoted $\mathbf{F}_{\mathrm{re}}$.

Also shown in Fig. 1 is a *stress-free* intermediate configuration $\mathcal{B}_{\mathrm{i}}$, which in general consists of a collection of disjoint neighbourhoods experiencing unconstrained growth from the intact $\mathcal{B}_{0}$ so as to maintain a stress-free condition. It should be emphasized, however, that $\mathcal{B}_{\mathrm{i}}$ is not in general uniquely defined. The transformation from $\mathcal{B}_{0}$ to $\mathcal{B}_{\mathrm{i}}$ is described by a so-called *growth tensor*, denoted $\mathbf{F}_{\mathrm{g}}$. The deformation gradient $\mathbf{F}$ is decomposed multiplicatively in the standard form introduced by Rodriguez et al. [44] as

2 $$ \mathbf{F} = \mathbf{F}_{\mathrm{e}} \mathbf{F}_{\mathrm{g}}, $$

where $\mathbf{F}_{\mathrm{e}}$ and $\mathbf{F}_{\mathrm{g}}$ are the elastic and growth parts of deformation gradient, respectively. Since the latter relates an intact configuration to a disjoint collection of neighbourhoods, it is not (in general) the gradient of a deformation. Similarly, $\mathbf{F}_{\mathrm{ie}}$ denotes the elastic deformation gradient (again not the gradient of a deformation) that takes $\mathcal{B}_{\mathrm{i}}$ to the unloaded configuration $\mathcal{B}_{\mathrm{r}}$, having the effect of gluing together the collection of incompatible pieces of the intermediate configuration by application of the residual stress.

From a phenomenological standpoint, $\mathbf{F}_{\mathrm{ie}}^{ - 1}$ may be interpreted as the deformation that releases the residual stress associated with the incompatibility of unconstrained growth from the corresponding neighbourhood in $\mathcal{B}_{\mathrm{r}}$, while $\mathbf{F}_{\mathrm{re}}$ is the elastic deformation gradient (an actual gradient of a deformation) from $\mathcal{B}_{\mathrm{r}}$ arising on application of the mechanical loads. Then, $\mathbf{F}_{\mathrm{e}}$ represents the combined effect of $\mathbf{F}_{\mathrm{ie}}$ and $\mathbf{F}_{\mathrm{re}}$ taking $\mathcal{B}_{\mathrm{i}}$ to ℬ, so that (locally)

3 $$ \mathbf{F}_{\mathrm{e}} = \mathbf{F}_{\mathrm{re}} \mathbf{F}_{ \mathrm{ie}}. $$

The deformation gradient relating $\mathcal{B}_{\mathrm{r}}$ to $\mathcal{B}_{0}$ is denoted $\mathbf{F}_{\mathrm{r}}$, with the decomposition

4 $$ \mathbf{F}_{\mathrm{r}} = \mathbf{F}_{\mathrm{ie}} \mathbf{F}_{\mathrm{g}}, $$

and it should be noted that $\mathbf{F}_{\mathrm{r}}$ is the gradient of a deformation, that which maps points of $\mathcal{B}_{0}$ smoothly and contiguously into those of the residually-stressed configuration $\mathcal{B}_{\mathrm{r}}$, without the need to make any reference to the fictitious (or virtual) configuration $\mathcal{B}_{\mathrm{i}}$. We can think of the material in $\mathcal{B}_{0}$ as growing into the configuration $\mathcal{B}_{\mathrm{r}}$ and developing residual stress. Of course, this doesn’t happen in practice because in general the growth actually takes place in the loaded configuration ℬ.

Based on the above discussion, the deformation gradient $\mathbf{F}$ relating ℬ to $\mathcal{B}_{0}$ may be decomposed as either

5 $$ \mathbf{F} = \mathbf{F}_{\mathrm{re}} \mathbf{F}_{\mathrm{r}}\quad \mbox{or}\quad \mathbf{F} = \mathbf{F}_{\mathrm{e}}\mathbf{F}_{\mathrm{g}}, $$

the latter being the kinematic growth decomposition (2) of Rodriguez et al. [44]. In the present paper we use the decomposition in (5)_1_ with the unloaded configuration as a starting point in order to explore the relationship between growth and residual stress.

The local volume ratios associated with the various deformation gradients are given by

6 $$\begin{aligned} J_{\mathrm{g}}=\det \mathbf{F}_{\mathrm{g}},\quad J_{\mathrm{ie}}=\det \mathbf{F}_{\mathrm{ie}}\quad J_{\mathrm{re}}=\det \mathbf{F}_{ \mathrm{re}},\quad J_{\mathrm{e}}=\det \mathbf{F}_{\mathrm{e}},\quad J_{\mathrm{r}}=\det \mathbf{F}_{\mathrm{r}}, \end{aligned}$$

and the mass densities in the configurations $\mathcal{B}_{0}$, $\mathcal{B}_{\mathrm{i}}$, $\mathcal{B}_{\mathrm{r}}$ and ℬ are denoted $\rho _{0}$, $\rho _{\mathrm{i}}$, $\rho _{\mathrm{r}}$ and $\rho $, respectively, with the connections

7 $$\begin{aligned} J =&J_{\mathrm{re}} J_{\mathrm{r}}=J_{\mathrm{re}} J_{\mathrm{ie}}J_{\mathrm{g}}=J_{\mathrm{e}} J_{\mathrm{g}}=\rho _{0}/\rho , \\ \rho _{0} =&J_{\mathrm{g}}\rho _{\mathrm{g}}=J_{\mathrm{r}}\rho _{\mathrm{r}}, \quad \rho _{\mathrm{r}}=J_{\mathrm{re}}\rho =\rho _{\mathrm{g}}/J_{ \mathrm{ie}}. \end{aligned}$$

It is noted that attention is restricted to volumetric growth, and for the moment no internal constraint on the material response, such as incompressibility, is imposed. However, if the elastic response of the material is incompressible, as will be considered later, then $J_{\mathrm{ie}}=J_{\mathrm{re}}=J_{\mathrm{e}}=1$, $J=J_{\mathrm{r}}=J_{\mathrm{g}}$, and $\rho =\rho _{\mathrm{r}}=\rho _{\mathrm{g}}$.

### Stress and Equilibrium

Let $\boldsymbol{\tau }$ denote the residual (Cauchy) stress in $\mathcal{B}_{\mathrm{r}}$, noting that, when evaluated in this configuration, there is no distinction between different measures of stress. The residually-stressed configuration is in equilibrium in the absence of mechanical loads, $\boldsymbol{\tau }$ is symmetric and satisfies the equilibrium equation and boundary condition

8 $$ \operatorname {Div}\boldsymbol{\tau } = \mathbf{0}\quad \mbox{in}\ \mathcal{B}_{\mathrm{r}}, \quad \boldsymbol{\tau } \mathbf{N}=\mathbf{0}\quad \mbox{on}\ \partial \mathcal{B}_{\mathrm{r}}, $$

where $\operatorname {Div}$ is the divergence operator with respect to $\mathcal{B}_{\mathrm{r}}$ and $\mathbf{N}$ is the unit outward normal to the boundary $\partial \mathcal{B}_{\mathrm{r}}$ of $\mathcal{B}_{\mathrm{r}}$. While the configuration $\mathcal{B}_{0}$ is fixed, the residually stressed configuration $\mathcal{B}_{\mathrm{r}}$ evolves with growth, as does the configuration $\mathcal{B}_{\mathrm{i}}$.

Let $\boldsymbol{\sigma }$ denote the Cauchy stress in configuration ℬ. Then, in the absence of body forces, it satisfies the equilibrium equation

9 $$ \operatorname {div}\boldsymbol{\sigma }=\mathbf{0}, $$

where $\operatorname {div}$ is the divergence operator with respect to ℬ. Note that inertia terms have been omitted since growth is a slowly evolving process and can be treated as quasi-static.

Unlike the intermediate configuration where the deformation gradient $\mathbf{F}_{\mathrm{g}}$ contains information relating to growth, the unloaded configuration involves the residual stress tensor $\boldsymbol{\tau } $, which is a function of $\mathbf{F}_{\mathrm{ie}}$ (and, possibly, any internal variables involved). But $\mathbf{F}_{\mathrm{ie}}$ depends on the growth that has taken place to reach the fictitious stress-free configuration $\mathcal{B}_{\mathrm{i}}$, and therefore $\boldsymbol{\tau }$ can be regarded as a proxy for growth (as also can $\mathbf{F}_{\mathrm{r}}$). This therefore provides a general platform for understanding the role of residual stresses in growth.

### Free Energy Functions and Constitutive Equations

Suppose that $\Psi $ is the free energy per unit mass associated with the open-system configuration ℬ, and that, as above, $\boldsymbol{\sigma }$ is the Cauchy stress, which satisfies the equilibrium equation (9). Then, by standard thermodynamic principles (see, e.g., [23]) the dissipation rate of the system, denoted $\mathcal{D}$, is given by

10 $$ \mathcal{D}= \boldsymbol{\sigma }:\mathbf{L} - \rho \hspace{1pt} \dot{\Psi }\ge 0, $$

where : denotes the double contraction, i.e., in terms of the trace, $\boldsymbol{\sigma }:\mathbf{L} =\operatorname {tr}(\boldsymbol{\sigma } \mathbf{L})$, and

11 $$ \mathbf{L} = \mathbf{\dot{F}}\mathbf{F}^{ - 1} $$

is the spatial velocity gradient, a superposed dot representing the material time derivative. In the context of the first-order theory of volumetric growth the constitutive constraint (10) was utilized by Epstein and Maugin [14] and Guillou and Ogden [22], for example.

Thermodynamic principles require that growth models and their evolution laws must satisfy (10). Thus, it should be possible to introduce, for example, as we shall do shortly, the residual stress $\boldsymbol{\tau }$ as a constitutive variable and its evolution equation so that the inequality (10) is satisfied without reference to the intermediate configuration $\mathcal{B}_{\mathrm{i}}$. However, in order to highlight the connection between the model to be presented and the kinematical growth model based on the decomposition (2), we begin the following discussion with $\mathbf{F}_{\mathrm{g}}$, and then link this to $\boldsymbol{\tau }$. Thus, the present method may give direct insight into the role of residual stress by regarding it as a growth variable rather than a consequence of growth.

Since mass is changing it is convenient to define the free energy per unit *fixed volume* in $\mathcal{B}_{0}$ rather than per unit mass. This is denoted $\Psi _{0}$ and given by

12 $$ \Psi _{0} = \rho _{0} \Psi , $$

where, we recall, $\rho _{0}=J\rho $ and $J=\det \mathbf{F}$. It should be emphasized that while the geometry of the configuration $\mathcal{B}_{0}$ is fixed the density $\rho _{0}$ within it changes with growth.

Henceforth, $\Psi _{0}$ is regarded as the fundamental free energy function, and its variants defined with respect to different configurations are discussed in the following. Thus, we have to consider on which variables $\Psi _{0}$ depends. To set the scene we begin with the standard formulation for which the free energy depends on $\mathbf{F}_{\mathrm{e}}$ and $\mathbf{F}_{\mathrm{g}}$, which requires knowledge of an inaccessible fictitious configuration. We then examine approaches in which the free energy depends on either $\mathbf{F}_{\mathrm{re}}$ and $\mathbf{F}_{\mathrm{r}}$ or $\mathbf{F}_{\mathrm{re}}$ and $\boldsymbol{\tau }$, both of which relate to a configuration for which experimental data can be obtained.

#### Approach 1: Free Energy Depending on $\mathbf{F}_{\mathrm{g}}$

We take $\Psi _{0}$ to depend on the growth through $\mathbf{F}_{\mathrm{g}}$ and separately on the total deformation through $\mathbf{F}$. Equivalently, since $\mathbf{F}=\mathbf{F}_{\mathrm{e}}\mathbf{F}_{\mathrm{g}}$, we can regard $\Psi _{0}$ as being dependent on $\mathbf{F}_{\mathrm{g}}$ and $\mathbf{F}_{\mathrm{e}}$. Thus, we write $\Psi _{0}(\mathbf{F}_{\mathrm{e}},\mathbf{F}_{\mathrm{g}})$. It is also possible to include internal variables, but for simplicity these are omitted in the present treatment.

We now introduce the notation $\Psi _{\mathrm{i}}$ for the free energy density per unit volume in the stress-free configuration $\mathcal{B}_{\mathrm{i}}$ via

13 $$ \Psi _{0} \left ( \mathbf{F}_{\mathrm{e}},\mathbf{F}_{\mathrm{g}} \right ) = J_{\mathrm{g}} \Psi _{\mathrm{i}} \left ( \mathbf{F}_{\mathrm{e}} \right ), $$

as in [2], but in different notation, so that $\Psi _{\mathrm{i}} =\rho _{\mathrm{g}}\Psi $. In general, $\Psi _{\mathrm{i}}$ can be expected to depend on the growth that has taken place, i.e. the material properties will be modified by the growth, which is commonly referred to as *remodelling*. Here, however, we are concerned only with volumetric growth so that the material response characterized by $\Psi _{\mathrm{i}}$ is independent of $\mathbf{F}_{\mathrm{g}}$, which is therefore omitted from the arguments of $\Psi _{\mathrm{i}}$. The factor $J_{\mathrm{g}}$ in front of $\Psi _{\mathrm{i}}$ reflects the (growth) volume change resulting from the change of reference configuration from $\mathcal{B}_{0}$ to $\mathcal{B}_{\mathrm{i}}$. We should also bear in mind the implications of objectivity, which requires that $\Psi _{\mathrm{i}}$ depends on $\mathbf{F}_{\mathrm{e}}$ through the right Cauchy–Green tensor $\mathbf{C}_{\mathrm{e}}=\mathbf{F}_{\mathrm{e}}^{\mathrm{T}}\mathbf{F}_{\mathrm{e}}$, where ^T^ signifies the transpose. We do not, however, write the $\mathbf{C}_{\mathrm{e}}$ explicitly as an argument of $\Psi _{\mathrm{i}}$, but take it as implicit.

Since we are considering volumetric growth, the density $\rho _{\mathrm{g}}$ is assumed not to change in time. Substitution of equations (12) and (13) into the inequality (10) and use of the assumption $\dot{\rho }_{\mathrm{g}}=0$ yields

14 $$ J\boldsymbol{\sigma }:\mathbf{L} - J_{\mathrm{g}} \dot{\Psi }_{\mathrm{i}} \geq 0. $$

In terms of $\Psi _{0}$ this can now be written

15 $$ J\boldsymbol{\sigma }:\mathbf{L} - \frac{\partial \Psi _{0}}{\partial \mathbf{F}_{\mathrm{e}}}: \mathbf{\dot{F}}_{\mathrm{e}} \geq 0 $$

by using the fact that $J_{\mathrm{g}}$ is assumed independent of $\mathbf{F}_{\mathrm{e}}$.

Next, by using the decomposition (2), we separate the spatial velocity gradient in the form

16 $$ \mathbf{L} = \mathbf{L}_{\mathrm{e}} + \mathbf{\bar{L}}_{\mathrm{g}}, $$

where, with reference to equation (11), the elastic and growth parts of $\mathbf{L}$ are defined respectively as $\mathbf{L}_{\mathrm{e}} = \mathbf{\dot{F}}_{\mathrm{e}} \mathbf{F}_{\mathrm{e}}^{ - 1}$ and $\mathbf{\bar{L}}_{\mathrm{g}} = \mathbf{F}_{\mathrm{e}}\mathbf{\dot{F}}_{\mathrm{g}} \mathbf{F}_{\mathrm{g}}^{ - 1} \mathbf{F}_{\mathrm{e}}^{ - 1}$, in which $\mathbf{\dot{F}}_{\mathrm{g}} \mathbf{F}_{\mathrm{g}}^{ - 1}$ is the growth velocity gradient $\mathbf{L}_{\mathrm{g}}$ in $\mathcal{B}_{\mathrm{i}}$. Substitution of the decomposition of $\mathbf{L}$ into the inequality (15) and use of the connection $J=\rho _{0}/\rho $ leads to

17 $$ \left ( J\boldsymbol{\sigma } - \mathbf{F}_{\mathrm{e}} \frac{\partial \Psi _{0}}{\partial \mathbf{F}_{\mathrm{e}}} \right ): \mathbf{L}_{\mathrm{e}} + J\boldsymbol{\sigma }:\mathbf{\bar{L}}_{\mathrm{g}} \geq 0. $$

Initially we are considering the elastic response not to be subject to any internal constraint and hence $\mathbf{L}_{\mathrm{e}} $ is arbitrary. Then, the inequality (17) allows us to deduce in a standard way (see, e.g., [23]) the elastic constitutive equation, in either of the equivalent forms

18 $$ \boldsymbol{\sigma } = J^{-1}\mathbf{F}_{\mathrm{e}} \frac{\partial \Psi _{0}}{\partial \mathbf{F}_{\mathrm{e}}}= J_{\mathrm{e}}^{-1}\mathbf{F}_{\mathrm{e}} \frac{\partial \Psi _{\mathrm{i}}}{\partial \mathbf{F}_{\mathrm{e}}}, $$

and the residual inequality

19 $$ \boldsymbol{\sigma }:\mathbf{\bar{L}}_{\mathrm{g}} \geq 0. $$

Note that the latter can also be written as $\mathbf{M}_{\mathrm{e}}:\mathbf{L}_{\mathrm{g}}\geq 0$, where $\mathbf{M}_{\mathrm{e}}$ is the Mandel stress, defined, in respect of $\mathbf{F}_{\mathrm{e}}$, by $\mathbf{M}_{\mathrm{e}}=J_{\mathrm{e}}\mathbf{F}_{\mathrm{e}}^{\mathrm{T}} \boldsymbol{\sigma }\mathbf{F}_{\mathrm{e}}^{-\mathrm{T}}$.

If we evaluate (18) in the configuration $\mathcal{B}_{\mathrm{r}}$, i.e., for $\mathbf{F}_{\mathrm{re}}=\mathbf{I}_{\mathrm{r}}$ (the value of $\mathbf{F}_{\mathrm{re}}$ in $\mathcal{B}_{\mathrm{r}}$) and $\mathbf{F}_{\mathrm{e}}=\mathbf{F}_{\mathrm{ie}}$, we obtain the (residual) Cauchy stress $\boldsymbol{\tau }$ in $\mathcal{B}_{\mathrm{r}}$. Thus,

20 $$ \boldsymbol{\tau } = J_{\mathrm{ie}}^{-1}\mathbf{F}_{\mathrm{ie}} \frac{\partial \Psi _{\mathrm{i}} }{\partial \mathbf{F}_{\mathrm{e}}}( \mathbf{F}_{\mathrm{ie}} ), $$

showing that $\boldsymbol{\tau }$ depends on $\mathbf{F}_{\mathrm{ie}}$.

By using the polar decomposition $\mathbf{F}_{\mathrm{ie}}=\mathbf{R}_{\mathrm{ie}}\mathbf{U}_{ \mathrm{ie}}$, where $\mathbf{R}_{\mathrm{ie}}$ is a rotation and $\mathbf{U}_{\mathrm{ie}}$ the right stretch tensor with $\mathbf{C}_{\mathrm{ie}}=\mathbf{U}_{\mathrm{ie}}^{2}$, we obtain

21 $$ \mathbf{R}_{\mathrm{ie}}^{\mathrm{T}}\boldsymbol{\tau }\mathbf{R}_{ \mathrm{ie}} = 2J_{\mathrm{ie}}^{-1}\mathbf{U}_{\mathrm{ie}} \frac{\partial \Psi _{\mathrm{i}} }{\partial \mathbf{C}_{\mathrm{e}}} \left ( \mathbf{F}_{\mathrm{ie}} \right )\mathbf{U}_{\mathrm{ie}}, $$

remembering that $\Psi _{\mathrm{i}}$ depends on $\mathbf{F}_{\mathrm{e}}$ only through $\mathbf{C}_{\mathrm{e}}$, (21) being evaluated for $\mathbf{F}_{\mathrm{e}}=\mathbf{F}_{\mathrm{ie}}$. Thus, when rotated with $\mathbf{R}_{\mathrm{ie}}$, $\boldsymbol{\tau }$ depends only on $\mathbf{U}_{\mathrm{ie}}$, equivalently on $\mathbf{C}_{\mathrm{ie}}$.

While $\Psi _{\mathrm{i}}$ is independent of the growth, i.e., it does not depend explicitly on $\mathbf{F}_{\mathrm{g}}$, $\mathbf{F}_{\mathrm{ie}}$ must depend on the growth since it is required to convert the incompatible (stress-free, grown) configuration $\mathcal{B}_{\mathrm{i}}$, which itself depends on $\mathbf{F}_{\mathrm{g}}$, into $\mathcal{B}_{\mathrm{r}}$. Thus, we can regard $\mathbf{F}_{\mathrm{ie}}$ as a function (more generally a functional) of $\mathbf{F}_{\mathrm{g}}$, and write

22 $$ \mathbf{F}_{\mathrm{ie}}=\boldsymbol{\mathcal{G}}(\mathbf{F}_{\mathrm{g}}), $$

where $\boldsymbol{\mathcal{G}}$ is a tensor function (or functional). Hence, $\boldsymbol{\tau }$ depends on $\mathbf{F}_{\mathrm{g}}$, thus showing that residual stress can be treated as a measure of growth. We can therefore formulate the growth problem by reference to residual stress in the unloaded configuration $\mathcal{B}_{\mathrm{r}}$ without the need to consider the artificial stress-free configuration, which can never be realized in practice. The residual stress, of course, evolves as growth proceeds, and therefore if it is adopted as a variable an evolution equation for $\boldsymbol{\tau }$ will be needed, as is the case for $\mathbf{F}_{\mathrm{g}}$. In the literature it is often assumed that $\mathbf{F}_{\mathrm{g}}$ is represented by a symmetric matrix, so that from the polar decomposition $\mathbf{F}_{\mathrm{g}}=\mathbf{R}_{\mathrm{g}}\mathbf{U}_{\mathrm{g}}$, the rotation tensor $\mathbf{R}_{\mathrm{g}}$ being represented by an identity matrix in $\mathcal{B}_{\mathrm{i}}$, with $\mathbf{U}_{\mathrm{g}}$ the (symmetric) right stretch tensor. While bearing this in mind, for theoretical purposes we retain $\mathbf{F}_{\mathrm{g}}$ in its general form, but invoke the specialization as appropriate.

We also have

23 $$ \mathbf{F}_{\mathrm{r}}=\mathbf{F}_{\mathrm{ie}}\mathbf{F}_{\mathrm{g}}= \boldsymbol{\mathcal{G}}(\mathbf{F}_{\mathrm{g}})\mathbf{F}_{\mathrm{g}} = \boldsymbol{\mathcal{H}}(\mathbf{F}_{\mathrm{g}}), $$

wherein the tensor function (or functional) $\boldsymbol{\mathcal{H}}$ is defined, so that $\mathbf{F}_{\mathrm{r}}$ also depends on $\mathbf{F}_{\mathrm{g}}$. Thus, we can treat the free energy function as dependent on $\mathbf{F}_{\mathrm{r}}$ instead of $\mathbf{F}_{\mathrm{g}}$ or, equivalently, on $\boldsymbol{\tau }$. Moreover, $\mathbf{F}_{\mathrm{r}}$ is the gradient of a deformation, that which takes $\mathcal{B}_{0}$ to $\mathcal{B}_{\mathrm{r}}$, and $\boldsymbol{\mathcal{H}}(\mathbf{F}_{\mathrm{g}})$ must be consistent with this requirement.

If the elastic response of the material is incompressible then (18) and (20) are replaced by

24 $$ \boldsymbol{\sigma } = \mathbf{F}_{\mathrm{e}} \frac{\partial \Psi _{\mathrm{i}}}{\partial \mathbf{F}_{\mathrm{e}}}-p \mathbf{I},\quad \boldsymbol{\tau } = \mathbf{F}_{\mathrm{ie}} \frac{\partial \Psi _{\mathrm{i}}}{\partial \mathbf{F}_{\mathrm{e}}} \left ( \mathbf{F}_{\mathrm{ie}} \right ) -p_{\mathrm{r}}\mathbf{I}_{\mathrm{r}}, $$

where $p$ is a Lagrange multiplier, $\mathbf{I}$ is the identity tensor in ℬ and $p_{\mathrm{r}}$ is the counterpart of $p$ in $\mathcal{B}_{\mathrm{r}}$. The form of the residual inequality (19) is unchanged.

#### Approach 2: Free Energy Depending on $\mathbf{F}_{\mathrm{r}}$

Without any reference to the configuration $\mathcal{B}_{\mathrm{i}}$, we now formulate the problem based on a free energy function defined by using $\mathbf{F}_{\mathrm{r}}$ instead of $\mathbf{F}_{\mathrm{g}}$, and we write the free energy per unit volume in $\mathcal{B}_{\mathrm{r}}$ as $\Psi _{\mathrm{r}}(\mathbf{F}_{\mathrm{re}},\mathbf{F}_{\mathrm{r}})$, so that

25 $$ \Psi _{\mathrm{r}}=\rho _{\mathrm{r}}\Psi . $$

For compressible materials, with $\mathbf{L}=\mathbf{\bar{L}}_{\mathrm{r}}+\mathbf{L}_{\mathrm{re}}$, where $\mathbf{\bar{L}}_{\mathrm{r}}=\mathbf{F}_{\mathrm{re}}\mathbf{L}_{\mathrm{r}}\mathbf{F}_{\mathrm{re}}^{-1}$, with $\mathbf{L}_{\mathrm{r}}=\mathbf{\dot{F}}_{\mathrm{r}}\mathbf{F}_{\mathrm{r}}^{-1}$ and $\mathbf{L}_{\mathrm{re}}=\mathbf{\dot{F}}_{\mathrm{re}}\mathbf{F}_{\mathrm{re}}^{-1}$, the inequality (10) yields

26 $$ \left (J_{\mathrm{re}}\boldsymbol{\sigma }-\mathbf{F}_{\mathrm{re}} \frac{\partial \Psi _{\mathrm{r}}}{\partial \mathbf{F}_{\mathrm{re}}} \right ):\mathbf{L}_{\mathrm{re}} +\left (J_{\mathrm{re}} \boldsymbol{\sigma }-\mathbf{F} \frac{\partial \Psi _{\mathrm{r}}}{\partial \mathbf{F}_{\mathrm{r}}} \mathbf{F}_{\mathrm{re}}^{-1}\right ):\mathbf{\bar{L}}_{\mathrm{r}} + \Psi _{\mathrm{r}} \frac{\dot{\rho }_{\mathrm{r}}}{\rho _{\mathrm{r}}}\geq 0, $$

which yields the constitutive equation

27 $$ \boldsymbol{\sigma }=J_{\mathrm{re}}^{-1}\mathbf{F}_{\mathrm{re}} \frac{\partial \Psi _{\mathrm{r}}}{\partial \mathbf{F}_{\mathrm{re}}} $$

and, on use of (27), the residual inequality

28 $$ \left (\mathbf{F}_{\mathrm{re}} \frac{\partial \Psi _{\mathrm{r}}}{\partial \mathbf{F}_{\mathrm{re}}}- \mathbf{F}_{\mathrm{r}} \frac{\partial \Psi _{\mathrm{r}}}{\partial \mathbf{F}_{\mathrm{r}}} \right ):\mathbf{\bar{L}}_{\mathrm{r}} +\Psi _{\mathrm{r}} \frac{\dot{\rho }_{\mathrm{r}}}{\rho _{\mathrm{r}}}\geq 0. $$

Note that if $\Psi $ is used instead of $\Psi _{\mathrm{r}}$ then the term in $\dot{\rho }_{\mathrm{r}}$ in (26) and (28) is omitted.

If we evaluate (27) in $\mathcal{B}_{\mathrm{r}}$ we obtain the (residual) stress $\boldsymbol{\tau }$ in $\mathcal{B}_{\mathrm{r}}$ as

29 $$ \boldsymbol{\tau }= \frac{\partial \Psi _{\mathrm{r}}}{\partial \mathbf{F}_{\mathrm{re}}} \left (\mathbf{I}_{\mathrm{r}}, \mathbf{F}_{\mathrm{r}} \right ), $$

which again shows that the residual stress depends on the growth, this time through $\mathbf{F}_{\mathrm{r}}$. Actually, $\Psi _{\mathrm{r}}$ depends on $\mathbf{F}_{\mathrm{r}}$ through the corresponding right Cauchy–Green tensor $\mathbf{F}_{\mathrm{r}}^{\mathrm{T}}\mathbf{F}_{\mathrm{r}}$. Because of this equivalence we can also regard the free energy as a function of $\boldsymbol{\tau }$ instead of $\mathbf{F}_{\mathrm{r}}$.

For an incompressible material we have $J_{\mathrm{re}}=1$ and $\dot{\rho }_{\mathrm{r}}=0$ and the constitutive equation (27) changes to

30 $$ \boldsymbol{\sigma }=\mathbf{F}_{\mathrm{re}} \frac{\partial \Psi _{\mathrm{r}}}{\partial \mathbf{F}_{\mathrm{re}}}-p \mathbf{I} $$

and (29) to

31 $$ \boldsymbol{\tau }= \frac{\partial \Psi _{\mathrm{r}}}{\partial \mathbf{F}_{\mathrm{re}}}( \mathbf{I}_{\mathrm{r}},\mathbf{F}_{\mathrm{r}})-p_{\mathrm{r}}\mathbf{I}_{\mathrm{r}}, $$

while the residual inequality becomes

32 $$ \left (\mathbf{F}_{\mathrm{re}} \frac{\partial \Psi _{\mathrm{r}}}{\partial \mathbf{F}_{\mathrm{re}}}-p \mathbf{I}-\mathbf{F}_{\mathrm{r}} \frac{\partial \Psi _{\mathrm{r}}}{\partial \mathbf{F}_{\mathrm{r}}} \right ):\mathbf{\bar{L}}_{\mathrm{r}}\geq 0. $$

This brings us to the third approach, which does not involve $\mathbf{F}_{\mathrm{r}}$.

#### Approach 3: Free Energy Depending on $\boldsymbol{\tau }$

Again considering a compressible elastic response, we now define the free energy per unit volume in $\mathcal{B}_{\mathrm{r}}$ as $\bar{\Psi }_{\mathrm{r}}(\mathbf{F}_{\mathrm{re}},\boldsymbol{\tau })$

Similar to the discussion in Approach 2, the inequality (10) for compressible materials yields

33 $$ \left (J_{\mathrm{re}}\boldsymbol{\sigma }-\mathbf{F}_{\mathrm{re}} \frac{\partial \bar{\Psi }_{\mathrm{r}}}{\partial \mathbf{F}_{\mathrm{re}}} \right ):\mathbf{L}_{\mathrm{re}} +J_{\mathrm{re}}\boldsymbol{\sigma }: \mathbf{\bar{L}}_{\mathrm{r}} - \frac{\partial \bar{\Psi }_{\mathrm{r}}}{\partial \boldsymbol{\tau }}: \boldsymbol{\dot{\tau }} +\bar{\Psi }_{\mathrm{r}} \frac{\dot{\rho }_{\mathrm{r}}}{\rho _{\mathrm{r}}} \geq 0. $$

This leads to an equation for the Cauchy stress $\boldsymbol{\sigma }$ in ℬ, i.e.,

34 $$ \boldsymbol{\sigma }=J_{\mathrm{re}}^{-1}\mathbf{F}_{\mathrm{re}} \frac{\partial \bar{\Psi }_{\mathrm{r}}}{\partial \mathbf{F}_{\mathrm{re}}} $$

and the residual inequality

35 $$ \left (\mathbf{F}_{\mathrm{re}} \frac{\partial \bar{\Psi }_{\mathrm{r}}}{\partial \mathbf{F}_{\mathrm{re}}} \right ):\mathbf{\bar{L}}_{\mathrm{r}} - \frac{\partial \bar{\Psi }_{\mathrm{r}}}{\partial \boldsymbol{\tau }}: \boldsymbol{\dot{\tau }}+\bar{\Psi }_{\mathrm{r}} \frac{\dot{\rho }_{\mathrm{r}}}{\rho _{\mathrm{r}}} \geq 0. $$

As with Approach 2, if $\Psi $ is used instead of $\bar{\Psi }_{\mathrm{r}}$ the term in $\dot{\rho }_{\mathrm{r}}$ is omitted.

When evaluated in $\mathcal{B}_{\mathrm{r}}$ equation (34) yields the following restriction:

36 $$ \boldsymbol{\tau }= \frac{\partial \bar{\Psi }_{\mathrm{r}}}{\partial \mathbf{F}_{\mathrm{re}}}( \mathbf{I}_{\mathrm{r}},\boldsymbol{\tau }). $$

It should be borne in mind that in this approach the tensor $\mathbf{F}_{\mathrm{g}}$ is not the fundamental growth variable and the intermediate configuration $\mathcal{B}_{\mathrm{i}}$, if of interest, can be defined in terms of the residual stress $\boldsymbol{\tau }$. According to equation (22), this is equivalent to defining a mapping $\mathbf{F}_{\mathrm{ie}}^{-1}$ (in general incompatible) from ${\mathcal{B}}_{\mathrm{r}}$ to $\mathcal{B}_{\mathrm{i}}$ that depends on $\boldsymbol{\tau }$.

Since the configuration $\mathcal{B}_{\mathrm{i}}$ is stress free, by taking $\mathbf{F}_{\mathrm{re}}=\mathbf{F}_{\mathrm{ie}}^{-1}$ and setting $\boldsymbol{\sigma }=\mathbf{O}$ we obtain, bearing in mind that $\mathbf{F}_{\mathrm{ie}}$ is not a compatible deformation, a pointwise connection between $\boldsymbol{\tau }$ and $\mathbf{F}_{\mathrm{ie}}$, i.e.,

37 $$ \frac{\partial \bar{\Psi }_{\mathrm{r}}}{\partial \mathbf{F}_{\mathrm{re}}} \left (\mathbf{F}_{\mathrm{ie}}^{-1},\boldsymbol{\tau }\right )= \mathbf{O}, $$

where $\mathbf{O}$ is the zero tensor. Then, given $\boldsymbol{\tau }$, $\mathbf{F}_{\mathrm{ie}}^{-1}$ is determined via the implicit equation above. Since, by (22), $\mathbf{F}_{\mathrm{ie}}$ depends on $\mathbf{F}_{\mathrm{g}}$, this provides an implicit connection between $\boldsymbol{\tau }$ and the growth tensor $\mathbf{F}_{\mathrm{g}}$.

In the case of an incompressible material, the counterpart of (34) is the constitutive equation

38 $$ \boldsymbol{\sigma }=\mathbf{F}_{\mathrm{re}} \frac{\partial \bar{\Psi }_{\mathrm{r}}}{\partial \mathbf{F}_{\mathrm{re}}} (\mathbf{F}_{\mathrm{re}},\boldsymbol{\tau })-p\mathbf{I}, $$

the residual inequality is

39 $$ \boldsymbol{\sigma }:\mathbf{\bar{L}}_{\mathrm{r}}- \frac{\partial \bar{\Psi }_{\mathrm{r}}}{\partial \boldsymbol{\tau }}: \boldsymbol{\dot{\tau }}\geq 0, $$

and (36) is replaced by

40 $$ \boldsymbol{\tau }= \frac{\partial \bar{\Psi }_{\mathrm{r}}}{\partial \mathbf{F}_{\mathrm{re}}} (\mathbf{I}_{\mathrm{r}},\boldsymbol{\tau })-p_{\mathrm{r}}\mathbf{I}_{\mathrm{r}}. $$

The incompressible counterpart of (37) is

41 $$ \frac{\partial \bar{\Psi }_{\mathrm{r}}}{\partial \mathbf{F}_{\mathrm{re}}} (\mathbf{F}_{\mathrm{ie}}^{-1},\boldsymbol{\tau })-p_{\mathrm{i}} \mathbf{F}_{\mathrm{ie}}=\mathbf{O}, $$

$p_{\mathrm{i}}$ being the value of $p$ in $\mathcal{B}_{\mathrm{i}}$.

#### Illustration of Approach 3: Growth of a Residually Stressed Material

In order to illustrate Approach 3, we now consider a simple example of an incompressible free energy function $\bar{\Psi }_{\mathrm{r}}(\mathbf{F}_{\mathrm{re}},\boldsymbol{\tau })$, which, because of objectivity, depends on $\mathbf{F}_{\mathrm{re}}$ through the right Cauchy–Green tensor, $\mathbf{C}_{\mathrm{re}}=\mathbf{F}_{\mathrm{re}}^{\mathrm{T}}\mathbf{F}_{\mathrm{re}}$ and the residual stress $\boldsymbol{\tau }$, which can be considered as a structure tensor. In general for an incompressible material, without any other structure, $\bar{\Psi }_{\mathrm{r}}$ is a function of nine invariants of $\mathbf{C}_{\mathrm{re}}$ and $\boldsymbol{\tau }$ (see, for example, [9, 45]). However, for simplicity of illustration, we consider a free energy function that is a function of just the three invariants defined by

42 $$\begin{aligned} I_{1} = \operatorname {tr}\mathbf{C}_{\mathrm{re}},\quad I_{4}=\operatorname {tr}\boldsymbol{\tau }, \quad I_{6} = \operatorname {tr}( \boldsymbol{\tau } \mathbf{C}_{\mathrm{re}} ), \end{aligned}$$

the first being an isotropic invariant appropriate for a material without residual stress, and the other two reflecting dependence on the residual stress. More specifically, we suppose that the dependence of $\bar{\Psi } _{\mathrm{r}}$ on these three invariants is given by

43 $$ \bar{\Psi } _{\mathrm{r}} (I_{1},I_{4},I_{6}) =\frac{1}{2}\mu \left ( I_{1} - 3 \right ) + \frac{1}{2}\left ( I_{6} - I_{4} \right ), $$

a special case of a model introduced in [45], where the constant $\mu \,>0$ is the shear modulus in the configuration $\mathcal{B}_{\mathrm{r}}$ in the absence of residual stress, and the final term is consistent with the requirements of the dependence of the energy function on the residual stress highlighted in [9, 45].

By substituting equation (43) into the constitutive law (38), the Cauchy stress is found to have the form

44 $$ \boldsymbol{\sigma }= \mu \mathbf{B}_{\mathrm{re}}+ \mathbf{F}_{\mathrm{re}}\boldsymbol{\tau }\mathbf{F}_{\mathrm{re}}^{\mathrm{T}}-p \mathbf{I}, $$

wherein the left Cauchy–Green tensor $\mathbf{B}_{\mathrm{re}}$ is defined as

45 $$ \mathbf{B}_{\mathrm{re}} = \mathbf{F}_{\mathrm{re}} \mathbf{F}_{\mathrm{re}}^{\mathrm{T}}, $$

and we note that $\boldsymbol{\sigma }=\boldsymbol{\tau }$ in $\mathcal{B}_{\mathrm{r}}$ provided $p_{\mathrm{r}}=\mu $.

Hence, with reference to equation (41), by replacing $\mathbf{F}_{\mathrm{re}}$ with $\mathbf{F}_{\mathrm{ie}}^{-1}$ (pointwise), equation (44) gives

46 $$ p_{\mathrm{i}} \mathbf{B}_{\mathrm{ie}} = \boldsymbol{\tau } +\mu \mathbf{I}_{\mathrm{i}}, $$

where $\mathbf{I}_{\mathrm{i}}$ is the identity tensor in $\mathcal{B}_{\mathrm{i}}$, and on taking the determinant with use of the incompressibility $\det \mathbf{B}_{\mathrm{ie}}=1$, we obtain

47 $$ p_{\mathrm{i}}^{3}=\det (\boldsymbol{\tau }+\mu \mathbf{I}_{\mathrm{i}}). $$

The latter equation determines $p_{\mathrm{i}}$ in terms of the residual stress and $\mu $, and $\mathbf{B}_{\mathrm{ie}}$ is then given by equation (46). Thus, for any given residual stress ${\boldsymbol{\tau }}$, the left Cauchy–Green tensor $\mathbf{B}_{\mathrm{ie}} $ can be obtained, which means the left stretch tensor $\mathbf{V}_{\mathrm{ie}}$ from the polar decomposition $\mathbf{F}_{\mathrm{ie}} =\mathbf{V}_{\mathrm{ie}}\mathbf{R}_{\mathrm{ie}}$, can be obtained but the rotational part $\mathbf{R}_{\mathrm{ie}} $ is not given by equation (46). However, bearing in mind that $\mathbf{F}_{\mathrm{r}}=\mathbf{F}_{\mathrm{ie}}\mathbf{F}_{\mathrm{g}}$ and the lack of uniqueness of $\mathcal{B}_{\mathrm{i}}$, the rotation $\mathbf{R}_{\mathrm{ie}} $ can be absorbed into and accounted for through $\mathbf{F}_{\mathrm{g}}$ with an appropriate growth law specification.

Thus, we obtain an explicit formula for $\mathbf{B}_{\mathrm{ie}}$, namely

48 $$ \mathbf{B}_{\mathrm{ie}}= \frac{\boldsymbol{\tau }+\mu \mathbf{I}_{\mathrm{i}}}{[\det (\boldsymbol{\tau }+\mu \mathbf{I}_{\mathrm{i}})]^{1/3}}. $$

Equation (22) then relates $\boldsymbol{\tau }$ to $\mathbf{F}_{\mathrm{g}}$.

## Application to the Growth of an Incompressible Elastic Spherical Shell

In this section we consider the spherically symmetric growth of a thick-walled spherical shell in order to illustrate the theory of the preceding section. The elastic response of the material is taken to be incompressible. In the reference configuration $\mathcal{B}_{0}$ the shell geometry is defined in terms of spherical polar coordinates $(R,\Theta ,\Phi )$ by

$$ 0< A\leq R\leq B, \quad 0\leq \Theta \leq \pi , \quad 0\leq \Phi \leq 2 \pi . $$

For a material point with position vector $\mathbf{X} \in \mathcal{B}_{0}$, relative to the centre of the shell, $|\mathbf{X}|=R$.

In terms of the spatial coordinate system $(r,\theta ,\phi )$, the geometry of the shell in $\mathcal{B}_{\mathrm{r}}$ after growth and elastic deformation is given by

$$ a\leq r\leq b, \quad 0\leq \theta \leq \pi , \quad 0\leq \phi \leq 2 \pi , $$

and the image $\mathbf{x}$ in $\mathcal{B}_{\mathrm{r}}$ of $\mathbf{X}\in \mathcal{B}_{0}$ is given by $\mathbf{x}=r\mathbf{X}/R$, with

49 $$ r=Rf(R), $$

where $f$ is an unknown function that depends on both the growth and the elastic deformation. Note that here we are taking $\mathbf{F}$ to coincide with $\mathbf{F}_{r}$.

The deformation gradient from $\mathcal{B}_{0}$ to $\mathcal{B}_{\mathrm{r}}$ is then obtained as

50 $$ \mathbf{F}_{\mathrm{r}}=\operatorname {Grad}\mathbf{x}=f(R)\mathbf{I}+ \frac{f^{\prime }(R)}{R}\mathbf{X}\otimes \mathbf{X}, $$

where a prime indicates the derivative with respect to $R$. This is symmetric and its principal axes coincide with the spherical polar axes. The corresponding principal stretches, $\lambda _{\mathrm{r1}}$, $\lambda _{\mathrm{r2}}$, $\lambda _{\mathrm{r3}}$ are then simply read off as

51 $$ \lambda _{\mathrm{r1}}=Rf^{\prime }(R)+f(R),\quad \lambda _{\mathrm{r2}}= \lambda _{\mathrm{r3}}=f(R)=r/R. $$

### Case Study 1: Relation Between $\mathbf{F}_{\mathrm{r}}$ and $\mathbf{F}_{\mathrm{g}}$ Based on the Formulation on $\mathcal{B}_{\mathrm{i}}$

Equation (23) relates $\mathbf{F}_{\mathrm{r}}$ to $\mathbf{F}_{\mathrm{g}}$ and directly bridges the gap from the formulation based on the stress-free configuration $\mathcal{B}_{\mathrm{i}}$ (in general incompatible) to the new formulation based on the residually-stressed configuration $\mathcal{B}_{\mathrm{r}}$. In the present context this becomes a scalar equation, written in the form $\lambda _{\mathrm{r2}}=\mathcal{H}(\lambda _{\mathrm{g}1},\lambda _{ \mathrm{}g2})$, which will be made explicit shortly, where $\lambda _{\mathrm{g}1}$ and $\lambda _{\mathrm{g}2}=\lambda _{\mathrm{g}3}$ are the principal stretches associated with the spherically symmetric growth.

The elastic response from $\mathcal{B}_{\mathrm{i}}$ to $\mathcal{B}_{\mathrm{r}}$ corresponds to that of an incompressible material, so that the volume of the shell between radii $a$ and $r$ in $\mathcal{B}_{\mathrm{r}}$ is

52 $$ \frac{4}{3}\pi (r^{3}-a^{3}). $$

Since the deformation gradient $\mathbf{F}_{\mathrm{g}}$ is in general incompatible, the corresponding volume in $\mathcal{B}_{\mathrm{i}}$ cannot be calculated with a similar formula. We denote it by $V(R)$, and calculate it by pulling back from $\mathcal{B}_{\mathrm{i}}$ to the initial (intact) configuration $\mathcal{B}_{0}$ with the Jacobian $J_{\mathrm{g}}$ to obtain

53 $$ V(R)=4\pi \int _{A}^{R} \xi ^{2}J_{\mathrm{g}}(\xi )\mathrm{d}\xi , $$

within which $J_{\mathrm{g}}=\lambda _{\mathrm{g}1}\lambda _{\mathrm{g}2}^{2}$, which in general is discontinuous.

By incompressibility these two volumes are equal, and hence

54 $$ r^{3}=a^{3} +\frac{3V(R)}{4\pi }. $$

The stretch $\lambda _{\mathrm{r2}}=r/R$ is then given by

55 $$ \lambda _{\mathrm{r2}}=\frac{1}{R}\left (a^{3} +\frac{3V(R)}{4\pi } \right )^{1/3}\equiv \mathcal{H}(\lambda _{\mathrm{g}1},\lambda _{ \mathrm{g}2}), $$

which defines the functional ℋ. Since $\lambda _{\mathrm{r2}}=f(R)$ and $\lambda _{\mathrm{r1}}=Rf'(R)+f(R)$, we obtain

56 $$ \lambda _{\mathrm{r1}}=\frac{V'(R)}{4\pi R^{2}\lambda _{\mathrm{r2}}^{2}}, \quad \mbox{equivalently}\quad \lambda _{\mathrm{r1}}\lambda _{\mathrm{r2}}^{2}=J_{\mathrm{g}}(R). $$

Thus, $\lambda _{\mathrm{r1}}$ and $\lambda _{\mathrm{r2}}$, and hence $\mathbf{F}_{\mathrm{r}}$ are determined in terms of $\mathbf{F}_{\mathrm{g}}$ when the growth law is specified.

Let $(\lambda _{\mathrm{ie}}^{-2},\lambda _{\mathrm{ie}},\lambda _{\mathrm{ie}})$ be the stretches associated with the deformation from $\mathcal{B}_{\mathrm{i}}$ to $\mathcal{B}_{\mathrm{r}}$. The connection $\mathbf{F}_{\mathrm{r}}=\mathbf{F}_{\mathrm{ie}}\mathbf{F}_{\mathrm{g}}$ specializes to $\lambda _{\mathrm{r2}}=\lambda _{\mathrm{ie}}\lambda _{\mathrm{g}2}$, so that

57 $$ \lambda _{\mathrm{ie}}=\frac{1}{R\lambda _{\mathrm{g}2}}\left (a^{3} + \frac{3V(R)}{4\pi }\right )^{1/3}. $$

While $\lambda _{\mathrm{g}1}$ and $\lambda _{\mathrm{g}2}$ are known once the growth law is specified, $a$ is as yet unknown. To determine $a$ we first need to obtain an expression for the residual stress in $\mathcal{B}_{\mathrm{r}}$ and then to ensure that it satisfies the required equilibrium equation and boundary conditions.

For an incompressible material the residual stress $\boldsymbol{\tau }$ is given by equation (24)_2_. For the present geometry $\mathbf{F}_{\mathrm{ie}}$ is diagonal, with the principal stretches identified above, and $\boldsymbol{\tau }$ is correspondingly diagonal. We denote its principal components by $\tau _{1}, \tau _{2}=\tau _{3}$. The free energy $\Psi _{\mathrm{i}}$ depends only on the stretch $\lambda _{\mathrm{ie}}$, and we represent this dependence as $\hat{\Psi }(\lambda _{\mathrm{ie}})$. It then follows that $\tau _{1}$ and $\tau _{2}$ are related by

58 $$ \tau _{2}-\tau _{1}=\frac{1}{2}\lambda _{\mathrm{ie}}\hat{\Psi }'( \lambda _{\mathrm{ie}}), $$

the prime here indicating differentiation with respect to $\lambda _{\mathrm{ie}}$. The equilibrium equation (8) for $\boldsymbol{\tau }$ reduces to the single component equation

59 $$ \frac{\mathrm{d}\tau _{1}}{\mathrm{d}r}+\frac{2}{r}(\tau _{1}-\tau _{2})=0, $$

and $\tau _{1}$ must satisfy the zero traction boundary conditions $\tau _{1}=0$ on $r=a$ and $r=b$, specializing (8)_2_.

By substituting (58) into (59), integrating with respect to $r$ and using the boundary conditions on $\tau _{1}$, we obtain

60 $$ \int _{a}^{b}\lambda _{\mathrm{ie}}\hat{\Psi }'(\lambda _{\mathrm{ie}}) \frac{\mathrm{d}r}{r}=0, $$

and we note that from (55) we have

61 $$ b=\left (a^{3} +\frac{3V(B)}{4\pi }\right )^{1/3} . $$

Hence $b$ depends on $a$, and equation (60) therefore determines $a$ in terms of the growth for a given free energy function.

In respect of (60) we note that if $\lambda _{\mathrm{ie}}=1$ in $[a,b]$ then it follows that $\hat{\Psi }'(1)=0$, and then from (58) and (59) that there is no residual stress. To satisfy (60) $\hat{\Psi }'(\lambda _{\mathrm{ie}})$ has to take both positive and negative values within $[a,b]$.

From (54) and (57) with $J_{\mathrm{g}}=\lambda _{\mathrm{g}1}\lambda _{\mathrm{g}2}^{2}$, we obtain

62 $$ \frac{\mathrm{d}r}{r}\left [1-(R\lambda _{\mathrm{g}2}'+\lambda _{ \mathrm{g}2})\frac{\lambda _{\mathrm{ie}}^{3}}{\lambda _{\mathrm{g}1}} \right ]=\frac{\mathrm{d}\lambda _{\mathrm{ie}}}{\lambda _{\mathrm{ie}}}, $$

where, considering $\lambda _{\mathrm{g}2}$ as a function of $R$, the prime signifies differentiation with respect to $R$. It follows, on changing the independent variable to $\lambda _{\mathrm{ie}}$, that (60) can be expressed in the form

63 $$ \int _{\lambda _{a}}^{\lambda _{b}} \frac{\hat{\Psi }'(\lambda _{\mathrm{ie}})}{(\lambda _{\mathrm{ie}}^{3}-G)} \mathrm{d}\lambda _{\mathrm{ie}}=0, $$

where $\lambda _{a}=a/A\lambda _{\mathrm{g}2}(A)$, $\lambda _{b}=b/B\lambda _{\mathrm{g}2}(B)$ and $G=\lambda _{\mathrm{g}1}/(R\lambda _{\mathrm{g}2}'+\lambda _{ \mathrm{g}2})$.

As a simple example we take $\hat{\Psi }$ to have the neo-Hookean form

64 $$ \hat{\Psi }(\lambda _{\mathrm{ie}})=\frac{1}{2}\mu (2\lambda _{\mathrm{ie}}^{2}+\lambda _{\mathrm{ie}}^{-4}-3), $$

where $\mu $ is a positive constant, in which case (63) has the form

65 $$ \int _{\lambda _{a}}^{\lambda _{b}}(\lambda _{\mathrm{ie}}^{-2}+ \lambda _{\mathrm{ie}}^{-5})\left ( \frac{\lambda _{\mathrm{ie}}^{3}-1}{\lambda _{\mathrm{ie}}^{3}-G}\right ) \mathrm{d}\lambda _{\mathrm{ie}}=0. $$

It follows that if $G$ is identically 1 then there is no non-trivial solution of the above integral. This is the case, for example, if $\lambda _{\mathrm{g}1}=\lambda _{\mathrm{g}2}=\mbox{constant}$. More generally, the sign of the integrand depends on the relative disposition of $G$ and 1, with $G$ depending on $R$ in general. If $\lambda _{\mathrm{ie}}^{3}$ lies between 1 and $G$, whether $G$ is less than or greater than 1, the integrand is negative, otherwise it is positive. A similar consideration applies to the more general $\hat{\Psi }$.

### Case Study 2: Relation Between $\mathbf{F}_{\mathrm{g}}$ and $\boldsymbol{\tau }$ Based on the Formulation on $\mathcal{B}_{\mathrm{r}}$

In contrast to Case study 1, we now consider the formulation based on the unloaded configuration $\mathcal{B}_{\mathrm{r}}$ with the residual stress $\boldsymbol{\tau }$ as the growth variable, and the free energy function $\bar{\Psi }_{\mathrm{r}}$ given by equation (43), and the Cauchy stress $\boldsymbol{\sigma }$ in ℬ by (44).

By taking $\mathbf{F}_{\mathrm{re}}=\mathbf{F}_{\mathrm{ie}}^{-1}$, i.e., by taking ℬ to be $\mathcal{B}_{\mathrm{i}}$, which is stress free (emphsizing that this is pointwise because of the incompatibility of $\mathbf{F}_{\mathrm{ie}}$), we again obtain

66 $$ \mu \mathbf{I}_{\mathrm{i}}+\boldsymbol{\tau }-p_{\mathrm{i}}\mathbf{B}_{\mathrm{ie}}=\mathbf{O}, $$

from which we obtain $p_{\mathrm{i}}^{3}=\det (\boldsymbol{\tau }+\mu \mathbf{I}_{\mathrm{i}})$, and equation (48) is recovered.

For the spherical shell with residual stresses $\tau _{1}$ and $\tau _{2}=\tau _{3}$ the circumferential stretch $\lambda _{\mathrm{ie}}$ identified in Case study 1 is the given by

67 $$ \lambda _{\mathrm{ie}}^{2}=\left (\frac{\tau _{2}+\mu }{\tau _{1}+\mu } \right )^{1/3}. $$

Once $\mathbf{F}_{\mathrm{ie}}$ is obtained by the above procedure, $\mathbf{F}_{\mathrm{g}}$ and hence $\mathbf{F}_{\mathrm{r}}=\mathbf{F}_{\mathrm{ie}}\mathbf{F}_{\mathrm{g}}$ can be evaluated. Firstly, we consider the simple situation in which $\mathbf{F}_{\mathrm{r}}$ is known. Bearing in mind that $\mathcal{B}_{\mathrm{r}}$ is a physically tenable configuration, from the point of view of experiment it is in principle possible that the residual stress $\boldsymbol{\tau }$ can be measured in a known $\mathcal{B}_{\mathrm{r}}$, so that $\mathbf{F}_{\mathrm{ie}}$ and $\mathbf{F}_{\mathrm{r}}$ are known. Then, $\mathbf{F}_{\mathrm{g}}$ is given by $\mathbf{F}_{\mathrm{g}}=\mathbf{F}_{\mathrm{ie}}^{-1}\mathbf{F}_{\mathrm{r}}$.

Secondly, if $\mathbf{F}_{\mathrm{r}}$ is assumed to be an unknown field it has to be a compatible deformation. With $\mathbf{F}_{\mathrm{ie}}^{-1}$ known, taking $\mathcal{B}_{\mathrm{r}}$ to $\mathcal{B}_{\mathrm{i}}$, we can think of $\mathbf{F}_{\mathrm{g}}^{-1}$ as having the role of glueing neighbouring pieces in $\mathcal{B}_{\mathrm{i}}$ together in $\mathcal{B}_{0}$ to retain its compatibility. For the spherical wall example, the compatibility condition can be deduced from equation (51) as

68 $$ \frac{\mathrm{d}\left (R\lambda _{2} \right )}{\mathrm{d}R}=\lambda _{1}, $$

or equivalently

69 $$ \frac{\mathrm{d}\left (R\lambda _{\mathrm{ie}}\lambda _{\mathrm{g}2} \right )}{\mathrm{d}R}= \lambda _{\mathrm{ie}}^{-2}\lambda _{\mathrm{g}1}, $$

with $\lambda _{\mathrm{ie}}$ known.

However, neither equation (68) nor (69) is sufficient on its own to determine two unknown stretches without further information. One option is to specify a relation between $\lambda _{\mathrm{g}1}$ and $\lambda _{\mathrm{g}2}$, in which case the first-order differential equation (69) can be solved to determine the growth stretches. Such an assumption, with the ratio $\lambda _{\mathrm{g}1}/\lambda _{\mathrm{g}2}$ being assumed constant is often adopted in kinematic growth analyses. Alternatively, this ratio or $G$ can be specified as a function of $R$. In either case, given the residual stress $\boldsymbol{\tau }$ as a growth variable, both $\mathbf{F}_{\mathrm{g}}$ and $\mathbf{F}_{\mathrm{r}}$ can be determined. With this provision, the two approaches to modelling growth are equivalent.

## Conclusions

Growth in nature is generally heterogeneous and anisotropic. Residual stresses are the widely accepted evidence of the mechanical regulation of the heterogeneity and anisotropy of growth. Residual stresses exist in unloaded configurations and can be calibrated by, for example, the ‘opening angle’ technique. In the present work we have explored connections between the approaches to the analysis of growth based on the unloaded residually-stressed configuration $\mathcal{B}_{\mathrm{r}}$ and the approach based on the fictitious stress-free intermediate configuration $\mathcal{B}_{\mathrm{i}}$.

Since the unloaded configuration and its (residual) stress are taken as explicit growth variables, the $\mathcal{B}_{\mathrm{r}}$-based approaches can be directly related to experimental observations such as the ‘opening angle’ and how this (and the residual stress) evolves with growth. As such, the heterogeneity and anisotropy of *growth* may be modelled more directly based on the experimental observations than with the $\mathcal{B}_{\mathrm{i}}$-based approach, which usually considers the heterogeneity and anisotropy of *material* rather than the characterization of the growth process itself, as exemplified in reference [11]. From the point of view of computational modelling, the residual stress in the $\mathcal{B}_{\mathrm{r}}$-based approaches then becomes a control variable rather than a computational result and can provide direct insight into the role of stress in growth regulation.

The growth of a spherically symmetric shell of elastically incompressible material has been studied in order to illustrate the different approaches. On the one hand, in respect of Case Study 1, given $\mathbf{F}_{\mathrm{g}}$, the process of obtaining the unloaded configuration $\mathcal{B}_{\mathrm{r}}$ and its residual stress has been demonstrated using the $\mathcal{B}_{\mathrm{i}}$-based approach. On the other hand, given the residual stress in $\mathcal{B}_{\mathrm{r}}$, the $\mathcal{B}_{\mathrm{r}}$-based approach has been used in Case Study 2 to evaluate $\mathbf{F}_{\mathrm{g}}$ (or equivalently $\mathcal{B}_{\mathrm{i}}$) to demonstrate the feasibility of this method. Thus, these case studies illustrate growth modelling based on either $\mathcal{B}_{\mathrm{i}}$ or $\mathcal{B}_{\mathrm{r}}$ and their connections and specific features.

The different approaches may have relative advantages and disadvantages from the point of view of constitutive and computational modelling. However, in summary, in the present study we have considered an approach to growth modelling formulated on the unloaded configuration with clear physical meaning and direct connections to experimental observations. An application of the finite element formulation of the residually-stressed based mathematical model to illustrate the evolution of residual stress is to be considered in a forthcoming paper, along with modelling more complicated growth phenomena than for a spherical shell. Our subsequent work will also include specific forms of free energy function and growth laws based on residual stress evolution and their assessment against the thermodynamic restrictions included herein.
